# Supplementary material for: Labeled oxytocin administered via the intranasal route reaches the brain in rhesus macaques
Source: Nat Commun. 2020 Jun 3;11:2783. doi: 10.1038/s41467-020-15942-1 (PMC7270110; doi:10.1038/s41467-020-15942-1)
Supplement: Supplementary file 2 — Reporting Summary [file 41467_2020_15942_MOESM2_ESM.pdf]

nature research

Corresponding author(s): Mary R. Lee

Last updated by author(s): Mar 12, 2020

Reporting Summary

Nature Research wishes to improve the reproducibility of the work that we publish. This form provides structure for consistency and transparency in reporting. For further information on Nature Research policies, see Authors & Referees and the Editorial Policy Checklist.

Statistics

For all statistical analyses, confirm that the following items are present in the figure legend, table legend, main text, or Methods section.

n/a	Confirmed	
☐	☒	The exact sample size (*n*) for each experimental group/condition, given as a discrete number and unit of measurement
☐	☒	A statement on whether measurements were taken from distinct samples or whether the same sample was measured repeatedly
☐	☒	The statistical test(s) used AND whether they are one- or two-sided<br>*Only common tests should be described solely by name; describe more complex techniques in the Methods section.*
☒	☐	A description of all covariates tested
☐	☒	A description of any assumptions or corrections, such as tests of normality and adjustment for multiple comparisons
☐	☒	A full description of the statistical parameters including central tendency (e.g. means) or other basic estimates (e.g. regression coefficient) AND variation (e.g. standard deviation) or associated estimates of uncertainty (e.g. confidence intervals)
☐	☒	For null hypothesis testing, the test statistic (e.g. *F*, *t*, *r*) with confidence intervals, effect sizes, degrees of freedom and *P* value noted<br>*Give P values as exact values whenever suitable.*
☒	☐	For Bayesian analysis, information on the choice of priors and Markov chain Monte Carlo settings
☒	☐	For hierarchical and complex designs, identification of the appropriate level for tests and full reporting of outcomes
☐	☒	Estimates of effect sizes (e.g. Cohen's *d*, Pearson's *r*), indicating how they were calculated

Our web collection on statistics for biologists contains articles on many of the points above.

Software and code

Policy information about availability of computer code

Data collection	N/A
Data analysis	Data analysis was conducted using SPSS

For manuscripts utilizing custom algorithms or software that are central to the research but not yet described in published literature, software must be made available to editors/reviewers. We strongly encourage code deposition in a community repository (e.g. GitHub). See the Nature Research guidelines for submitting code & software for further information.

Data

Policy information about availability of data

All manuscripts must include a data availability statement. This statement should provide the following information, where applicable:

- Accession codes, unique identifiers, or web links for publicly available datasets
- A list of figures that have associated raw data
- A description of any restrictions on data availability

All raw data is included in Figures 1,2,3

Field-specific reporting

Please select the one below that is the best fit for your research. If you are not sure, read the appropriate sections before making your selection.

☒ Life sciences ☐ Behavioural & social sciences ☐ Ecological, evolutionary & environmental sciences

For a reference copy of the document with all sections, see nature.com/documents/nr-reporting-summary-flat.pdf

Life sciences study design

All studies must disclose on these points even when the disclosure is negative.

Sample size	This study built on a previous nonhuman primate study (n=6) (Lee et al., Molecular Psychiatry, 2018) measuring cerebrospinal fluid (CSF)concentrations of administered labelled oxytocin. This study extended that study by measuring brain concentrations in the same number of nonhuman primates (n=6) in a terminal experiment.
Data exclusions	No data were excluded from this study.
Replication	This was a terminal study in nonhuman primates, therefore, a second experiment to reproduce results from the first study was not able to be conducted.
Randomization	This was a repeated measures study where all animals received each dose-route combination of labelled oxytocin in a counterbalanced fashion. The six animals were then divided into groups of 2 for the terminal experiment as explained in the Methods section
Blinding	Blinding was not relevant to this study as it was conducted in nonhuman primates and outcomes were concentrations of labelled and endogenous oxytocin in plasma, CSF and brain.

Reporting for specific materials, systems and methods

We require information from authors about some types of materials, experimental systems and methods used in many studies. Here, indicate whether each material, system or method listed is relevant to your study. If you are not sure if a list item applies to your research, read the appropriate section before selecting a response.

Materials & experimental systems

n/a	Involved in the study
☒	☐ Antibodies
☒	☐ Eukaryotic cell lines
☒	☐ Palaeontology
☐	☒ Animals and other organisms
☒	☐ Human research participants
☒	☐ Clinical data

Methods

n/a	Involved in the study
☒	☐ ChIP-seq
☒	☐ Flow cytometry
☒	☐ MRI-based neuroimaging

Animals and other organisms

Policy information about studies involving animals; ARRIVE guidelines recommended for reporting animal research

Laboratory animals	See Table 1 of manusript for characteristics of each nonhuman primate (Rhesus Macaques)
Wild animals	Study did not involve wild animals
Field-collected samples	Study did not involve samples from the field
Ethics oversight	Study was reviewed and approved by the National Insititute on Alcohol Abuse and Alcoholism Animal Care and Use Committee (ACUC)

Note that full information on the approval of the study protocol must also be provided in the manuscript.
